# Mean platelet volume/platelet count ratio in combination with tumor markers in colorectal cancer: a retrospective clinical study

**DOI:** 10.1186/s12885-023-10585-z

**Published:** 2023-02-07

**Authors:** Huan Zhang, Fan Lin, Zhuocai Wang

**Affiliations:** 1 Department of Radiotherapy, General Hospital of Southern Theater Command, 510010 Guangzhou, China; 2 Department of Gastroenterology, General Hospital of Southern Theater Command, Guangzhou, China; 3 Department of Pathology, General Hospital of Southern Theater Command, Guangzhou, China

**Keywords:** Colorectal cancer, Tumor markers, Mean platelet volume/platelet count ratio, Combined detection

## Abstract

**Background:**

Mean platelet volume (MPV) is a marker of platelet activation, which is usually negatively correlated with platelet count (PC). The ratio of MPV to PC (MPV/PC) has an essential role in the diagnosis of multiple malignancies. However, only a few studies investigated the value of MPV/PC in colorectal cancer (CRC) and the combination of MPV/PC with tumor markers in CRC. This retrospective clinical study aimed to evaluate the diagnostic value of MPV/PC and tumor markers (CA72-4, CA125, CA199) used alone or in combination in CRC.

**Methods:**

200 patients with CRC and 317 patients with colorectal benign polypus pathologically diagnosed during 2019/01/04 to 2022/06/30 were included. Hematological and pathological parameters of the above patients were collected, data were analyzed with Student’s t-test, one-way ANOVA or Kruskal-Wallis H test and receiver operating characteristic (ROC) curve, and ROC curve was used to evaluate the diagnostic value of tumor markers and MPV/PC used alone or in combination in CRC.

**Results:**

The MPV/PC in CRC group was significantly lower than the control group (P < 0.0001). Among the three tumor markers, higher CA125 was correlated with distant metastasis and lower differentiation (P < 0.05), increased CA72-4 indicated positive nerve invasion (P = 0.0174), and elevated CA199 was associated with lymphatic metastasis and positive vascular invasion (P < 0.05). For subgroups regarding tumor anatomical location, both CA125 and CA199 were higher in colon cancer group than rectum cancer group (P = 0.0322, P = 0.0094). MPV/PC was associated with tumor infiltration, regional lymph node metastasis, differentiation and nerve invasion (P < 0.05) and the combination of MPV/PC with the three tumor markers produced a larger AUC with higher sensitivity, specificity and Yuden index than MPV/PC or the three tumor markers used alone to distinguish between CRC and colorectal polyps.

**Conclusion:**

Preoperative MPV/PC in peripheral blood of patients with CRC was lower than the control group. Meanwhile, the combined detection of tumor markers with MPV/PC can improve the diagnostic value of CRC, revealing the potential of MPV/PC as a promising screening tool in CRC early diagnosis.

## Introduction

Colorectal cancer (CRC) is the most common gastrointestinal malignant tumor with enormous disease and economic burden, ranking third in incidence and second in mortality rate [1]. CRC can remain clinically silent for years. When present, symptoms often develop insidiously over a period of months and years. Therefore, a majority of patients are diagnosed in the advanced stage, and lost the opportunity to receive the most appropriate treatment [2]. So far, coloscopy is regarded as the gold standard for the diagnosis of CRC [3]. However, the limitations such as uncomfortable experience, expensive cost and complications restrict its widespread application. Other screening methods also have some defects related to screening effectiveness, sensitivity and specificity [4]. Therefore, discovering simple, non-invasive, affordable and highly acceptable indicators is of great significance to improve the screening efficacy of CRC.

Tumor markers are a series of bioactive substances produced and secreted by malignant tumor cells in pathological processes of proliferation, invasion and metastasis, which are widely used for the early diagnosis and therapeutic effect monitoring of various tumors [5]. For example, carcinoembryonic antigen, carbohydrate antigen 72 − 4 (CA72-4), CA19-9 and CA125 are tumor markers commonly used in clinical practice for the detection and monitoring of CRC [6]. There are multiple tumor markers in the circulation. Different types of malignant tumors can produce the same tumor marker. Besides, one type of malignant tumor can produce multiple tumor markers. Moreover, not all tumor markers can be applied in clinical practice. Therefore, if only one type of tumor marker is used alone to detect cancer, clinicians may not account for specificity, sensitivity and repeatability. We speculated that the combined detection of multiple tumor markers may improve the diagnostic efficacy of malignant tumors.

Accumulating evidence has indicated that inflammation plays an essential role in the development and progression of various cancers, including CRC [7, 8]. Systematic inflammation status may be reflected by various markers, such as neutrophil-to-lymphocyte ratio (NLR), platelet-to-lymphocyte ratio (PLR), C-reactive protein, mean platelet volume (MPV), etc. [9, 10]. The above inflammatory markers may predict treatment response and progression of numerous cancers [11, 12]. Platelets were not only associated with hemostasis and thrombosis, but also participated in the process of tumor growth and metastasis [13]. MPV reflects the size of platelets in the circulation and the function and activity of the platelet [14]. Studies have demonstrated that MPV is a potential biomarker for the diagnosis and follow-up of cancers, and reduced MPV is associated with poor prognosis of patients with thyroid cancer or other tumors [15]. Given that MPV is negatively associated with platelet count (PC), Wu et al. proved that the MPV/PC ratio was better than the two variables used alone in the diagnosis of CRC [16]. Furthermore, multiple studies have explored the clinical value of the MPV/PC ratio in other malignant cancers and diseases [17].

At present, only a few studies have examined the relationship between MPV/PC and CRC and the value of MPV/PC combined with tumor markers in the diagnosis of CRC. The aim of this study was to investigate the individual and combined diagnostic value of preoperative CA72-4, CA125, CA199 and MPV/PC in newly diagnosed CRC patients and patients with benign colorectal polyps. To the best of our knowledge, this is the first study to investigate the combination of CA72-4, CA125 and CA199 with MPV/PC in the diagnosis of CRC.

## Methods

### Patients

In this retrospectively study, 200 patients who underwent surgical resection and were pathologically diagnosed with CRC in the Department of Gastrointestinal Surgery of the Southern Theater General Hospital during 2019/01/04 to 2022/06/30 were selected as the CRC group, and 317 patients diagnosed with colorectal benign polypus during the same period were selected as the control group. Histological diagnosis of CRC or colorectal polyps was later confirmed after surgical treatment or obtained from colonoscopy histology reports. There was no significant difference in gender or age between the two groups. All those patient’s disease history, family history, other basic data and laboratory examination data were detailed. The established study exclusion criteria were as follows: (a) recurrent CRC or 5-year history of another malignancy; (b) previous treatment with chemotherapy and/or radiotherapy; (c) evidence of other gastrointestinal, inflammatory, hematologic, hepatobiliary, pulmonary, and cardiovascular diseases; and (d) treatment with anti-aggregation and/or anticoagulant therapy, antilipemic therapy, and nonsteroidal anti-inflammatory drugs as well as recent blood transfusions. Tumor markers ( CA72-4, CA125, CA199), mean platelet volume (MPV) and platelet count (PC or PLT) before surgery or gastroenteroscopy were collected. Besides, other clinical data such as age, gender and pathological parameters tumor size, lymphatic metastasis, distant metastasis and differentiation were recorded. All CRC patients were staged according to the 8th edition of the United States Joint Cancer Committee (UICC/AJCC) TNM staging criteria. The study was approved by the Ethics Committee of the General Hospital of Southern Theater Command. Due to the nature of the retrospective study, the requirement of informed consent was waived by the Ethics Committee of the General Hospital of Southern Theater Command. 

### Clinical measurements and calculation

Fasting venous blood was taken into an ethylenediaminetetraacetic acid-K2 anticoagulant tube and a dry tube early during the early morning to separate the serum, and the three tumor markers were detected by electrochemiluminescence method according to the instructions by Roche e601 automatic electrochemiluminescence immunoanalyzer and the matching kit. A Beckman 780 blood cell analyzer (Beckman Coulter, Brea, CA) was used for the routine examination of blood samples. MPV/PC values were calculated from the mean platelet volume and the platelet count.

### Statistical analyses

The continuous variable data are expressed as mean ± standard deviation or median (interquartile range), and the categorical variable data are expressed in terms of frequency. All data were statistically analyzed using the software programs SPSS 27.0.1 (IBM, Armonk, NY, USA), MedCalc 15.0 (MedCalc Software, Mariakerke, Belgium) and Prism 5 (GraphPad Software, San Diego, CA, USA). Data were compared between two groups by Student’s *t*-test and three groups by one-way ANOVA or Kruskal-Wallis H test. Tumor markers and MPV/PC data were analyzed by Logistic regression analysis. The receiver operating characteristic (ROC) curve was used to calculate the sensitivity, specificity and area under the curve (AUC), and to evaluate the diagnostic values of the three tumor markers and MPV/PC used alone or in combination in CRC diagnosis. Statistical significance was defined as *P* < 0.05.

## Results

### Baseline characteristics of the enrolled participants

**Fig. 1:**
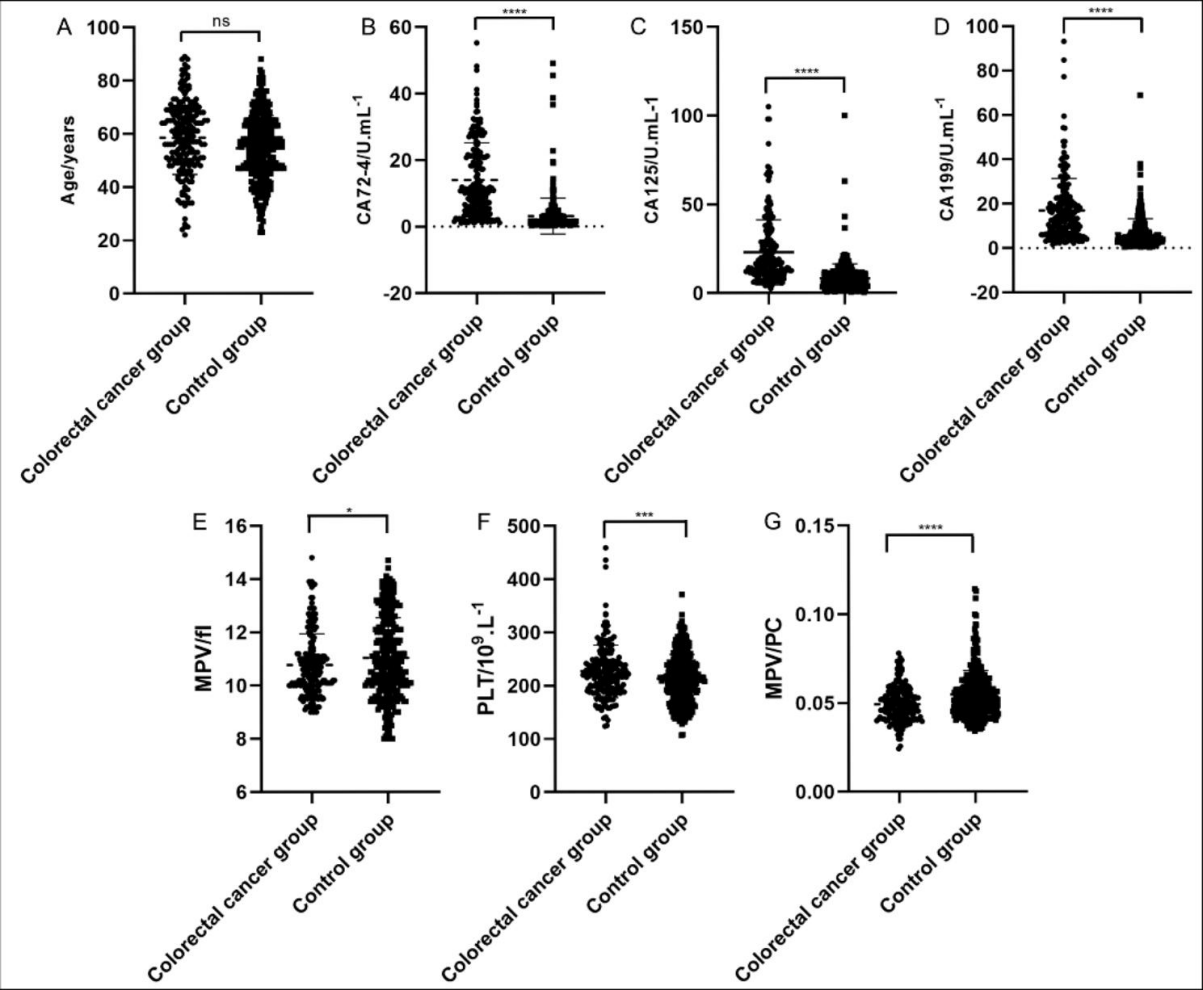
Clinical parameters of colorectal cancer group and colorectal polyps group. A. Ages of patients in colorectal cancer group and control group; B-G CA72-4, CA125, CA199, MPV, Platelet count and MPV/PC in colorectal cancer group and control group

From 2019/01/04 to 2022/06/30, 200 eligible patients with CRC were enrolled, including 128 males (64%) and 72 females (36%), aged 22 to 89 years old, median age 59 years old and average age 58.58 ± 13.75 years old. There were 317 eligible control group patients, including 212 males (66.88%) and 105 females (33.12%), aged 23 to 88 years old, median age 55 years old and average age 57.56 ± 12.31 years old. Compared with the control group, CA72-4, CA125, CA199 and platelet count were all significantly higher (all *P* < 0.001). However, MPV and MPV/PC both were lower than the control group, and the difference was statistically significant (*P* < 0.05) (Table 1; Fig. 1).

**Table 1:** Clinical parameters of colorectal cancer group and colorectal benign polyps group/$\stackrel{-}{x}$±*s*

Items	Colorectal cancer group (n = 200)	Control group (n = 317)	*P*
Age (years)	58.58 ± 13.75	57.56 ± 12.31	0.0660
CA72-4 (U/mL)	14.03 ± 11.09	3.17 ± 5.39	< 0.0001^****^
CA125 (U/mL)	23.04 ± 18.27	8.36 ± 8.05	< 0.0001^****^
CA199 (U/mL)	16.94 ± 14.39	6.53 ± 6.73	< 0.0001^****^
PLT (10^9^/L)	227.13 ± 49.15	213.08 ± 45.48	0.0008^***^
MPV (fl.)	10.77 ± 1.17	11.04 ± 1.51	0.0339^*^
MPV/PC	0.0493 ± 0.0099	0.0540 ± 0.0131	< 0.0001^****^

PLT, platelet; MPV, mean platelet volume. * *P* ≤ 0.05; *** *P* ≤ 0.001; **** *P* ≤ 0.0001

### Comparison of CA72-4, CA125, CA199 and MPV/PC among subgroups of colorectal cancer

The clinicopathological features of CRC subgroups and their preoperative values of tumor markers and MPV/PC are shown in Table 2. No statistically significant difference was found in CA72-4, CA125 and CA199 levels and the MPV/PC ratio in terms of sex and age (*P* > 0.05). Similarly, no significant differences were observed in CA72-4 in terms of TNM stage, differentiation, vascular invasion and tumor location. However, preoperative CA72-4 levels were higher in CRC patients with nerve invasion than in those without nerve invasion (*P* = 0.0174). Preoperative CA125 levels were higher in patients with stage T3-4, distant metastasis or poorly differentiated CRC (*P* < 0.05). Moreover, preoperative CA199 levels were higher in CRC patients with regional lymph node metastasis or vascular invasion (*P* < 0.05). Previous studies found that clinical and pathological features of CRC originating from different primary sites were distinct [18]. The present study found that preoperative tumor markers were different between patients with colon cancer and rectal cancer. Preoperative CA125 and CA199 levels in patients with colon cancer were 24.51 ± 15.04 and 18.71 ± 15.87 U/mL respectively, which were both higher than those of patients with rectum cancer (*P *= 0.0322, *P* = 0.0094). However, no significant difference was found in CA72-4 levels in terms of cancer location (*P* > 0.05). While MPV/PC did not significantly differ between patients with and without distant metastasis, it significantly differed in T-stage and regional lymph node metastasis subgroups. CRC patients with T3-4 stage, lymph node metastasis, poorly differentiated or nerve invasion had a lower preoperative MPV/PC ratio of 0.0480 ± 0.0096, 0.0474 ± 0.0079, 0.0478 ± 0.0098 and 0.0458 ± 0.0105 respectively (*P* = 0.0007, 0.0079, 0.0431 and 0.0013, respectively). The aforementioned subgroup indicators demonstrated malignant behavior, we therefore guessed that a lower preoperative MPV/PC ratio may be a promising marker for the diagnosis and prognosis prediction of CRC.

**Table 2:** Difference of CA72-4, CA125, CA199 and MPV/PC in the subgroups of colorectal cancer group/x ®±s

Items	N	CA72-4/U.mL^− 1^	*P*	CA125/U.mL^− 1^	*P*	CA199/ U.mL^− 1^	*P*	MPV/PC	*P*
**Age/years**									
≤ 59	101	13.53 ± 10.62	0.5230	22.35 ± 14.88	0.0651	17.63 ± 14.50	0.4978	0.0493 ± 0.0104	0.8142
>59	99	14.54 ± 11.53		23.80 ± 18.81		16.24 ± 12.24		0.0490 ± 0.0090	
**Gender**									
Male	128	14.29 ± 11.26	0.6601	22.41 ± 18.07	0.5121	15.54 ± 13.76	0.0667	0.0491 ± 0.0094	0.8394
Female	72	13.57 ± 10.76		24.18 ± 18.55		19.44 ± 15.13		0.0493 ± 0.0103	
**T stage**									
T1-2	48	12.44 ± 9.67	0.2556	18.80 ± 12.44	0.0451^*^	14.86 ± 11.44	0.2513	0.0534 ± 0.0090	0.0007^***^
T3-4	152	14.54 ± 11.46		24.39 ± 13.56		17.60 ± 15.15		0.0480 ± 0.0096	
**N stage**									
N0	96	13.58 ± 10.75	0.5849	22.03 ± 16.00	0.4506	14.34 ± 11.12	0.0137^*^	0.0511 ± 0.0106	0.0079^**^
N1-2	104	14.45 ± 11.38		23.99 ± 20.08		19.35 ± 16.50		0.0474 ± 0.0079	
**M stage**									
M0	166	13.85 ± 10.87	0.6035	21.57 ± 16.44	0.0116^*^	16.84 ± 14.36	0.8295	0.0494 ± 0.0100	0.4597
M1	34	14.94 ± 12.07		30.23 ± 24.11		17.43 ± 14.51		0.0480 ± 0.0083	
**Differentiation**									
Low	36	14.51 ± 11.32	0.7752	29.28 ± 23.81	0.0236^*^	19.98 ± 16.85	0.1640	0.0478 ± 0.0098	0.0431^*^
Mild-High	164	13.93 ± 11.04		21.68 ± 16.49		16.28 ± 3.70		0.0520 ± 0.0092	
**Location**									
Colon	132	14.36 ± 11.26	0.5348	24.51 ± 15.04	0.0322^*^	18.71 ± 15.87	0.0094^**^	0.0493 ± 0.0101	0.6790
Rectum	62	13.30 ± 10.66		19.79 ± 12.93		13.01 ± 9.22		0.0487 ± 0.0088	
**Nerve invasion**									
Positive	62	16.81 ± 12.09	0.0174^*^	26.29 ± 19.38	0.0934	17.86 ± 16.39	0.5464	0.0458 ± 0.0105	0.0013^**^
Negative	138	12.78 ± 10.37		21.59 ± 17.55		16.53 ± 13.38		0.0508 ± 0.0095	
**Vascularinvasion**									
Positive	47	15.45 ± 11.37	0.3192	24.70 ± 15.32	0.4806	22.26 ± 15.21	0.0041^**^	0.0501 ± 0.0100	0.4545
Negative	153	13.60 ± 10.97		22.54 ± 19.05		15.96 ± 12.16		0.0489 ± 0.0096	

Note. * *P* ≤ 0.05; ** *P* ≤ 0.01; *** *P* ≤ 0.001

### Diagnostic values of MPV/PC and tumor markers used alone for distinguishing between colorectal cancer and adenomatous polyps

**Fig. 2:**
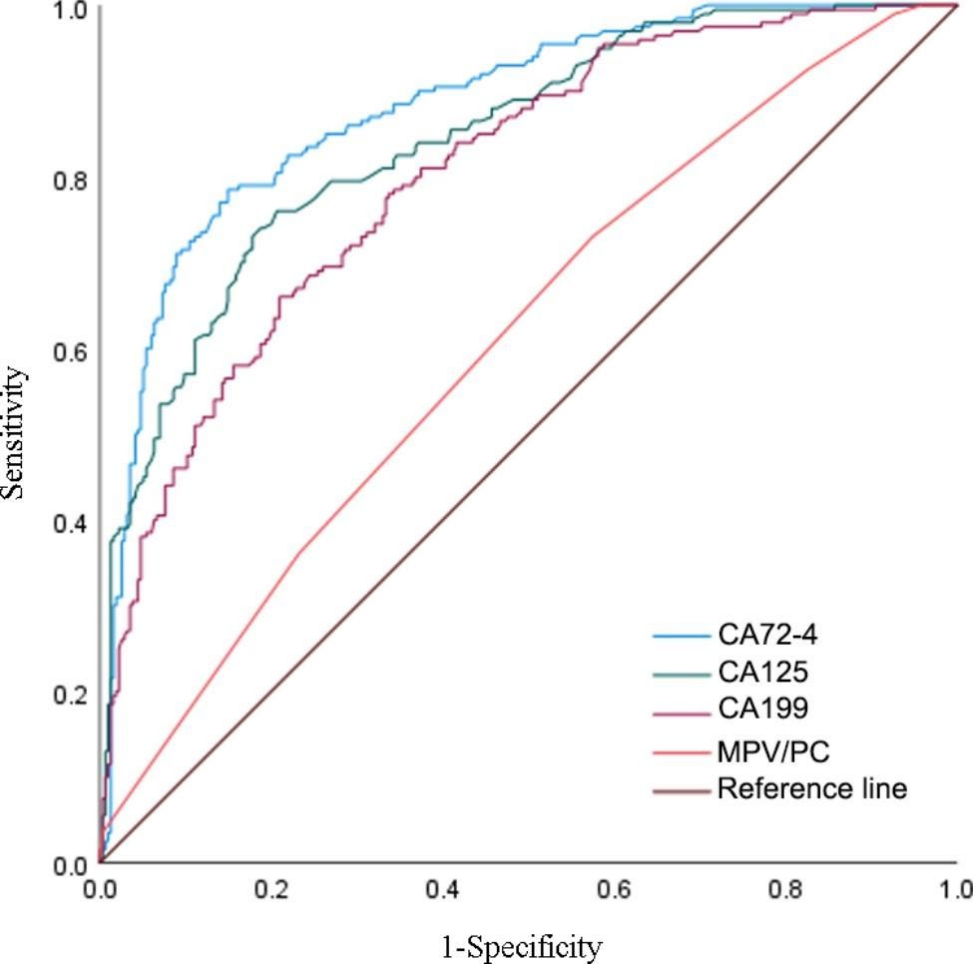
ROC curve when the three tumor markers and MPV/PC used alone to diagnose CRC.

The ROC curve was used to detect the diagnostic values of MPV/PC and tumor markers used alone for CRC. As shown in Table 3; Fig. 2, with the maximum Youden index as the cut-off point, the optimum cut-off value of CA72-4, CA125, CA199 and MPV/PC for diagnosing CRC was respectively 0.636, 0.554, 0.450 and 0.415. When the three tumor markers and MPV/PC were applied alone to diagnose CRC, the AUC of CA72-4 was 0.882, which was the highest of the four indicators, followed by CA125, CA199, and MPV/PC (with an AUC of 0.844, 0.803 and 0.610 respectively). The sensitivity order of the four indexes used alone to diagnose CRC was CA72-4 > CA125 > MPV/PC > CA199 (0.785 > 0.760 > 0.730 > 0.660), and the specificity order was CA72-4 > CA125 > CA199 > MPV/PC (0.851 > 0.794 > 0.790 > 0.685). In addition to the Youden index, the AUC, sensitivity, specificity, PPV and NPV of CA72-4 were all the highest of the four indicators, demonstrating that the diagnostic efficacy of CA72-4 in CRC was relatively high among the other three markers.

**Table 3:** Diagnostic performances of the three tumor markers and MPV/PC used alone for distinguishing colorectal cancer from benign colorectal polyps

Items	AUC(95%CI)	sensitivity	specificity	Youden index	PPV	NPV
CA72-4	0.882(0.852–0.912)	0.785	0.851	0.636	0.769	0.863
CA125	0.844(0.810–0.878)	0.760	0.794	0.554	0.699	0.840
CA199	0.803(0.765–0.841)	0.660	0.790	0.450	0.665	0.786
MPV/PC	0.610(0.561–0.659)	0.730	0.685	0.415	0.594	0.801

PPV positive predictive value, NPV negative predictive value

### Diagnostic values of MPV/PC in combination with tumor markers for differentiating between colorectal cancer and adenomatous polyps

**Fig. 3:**
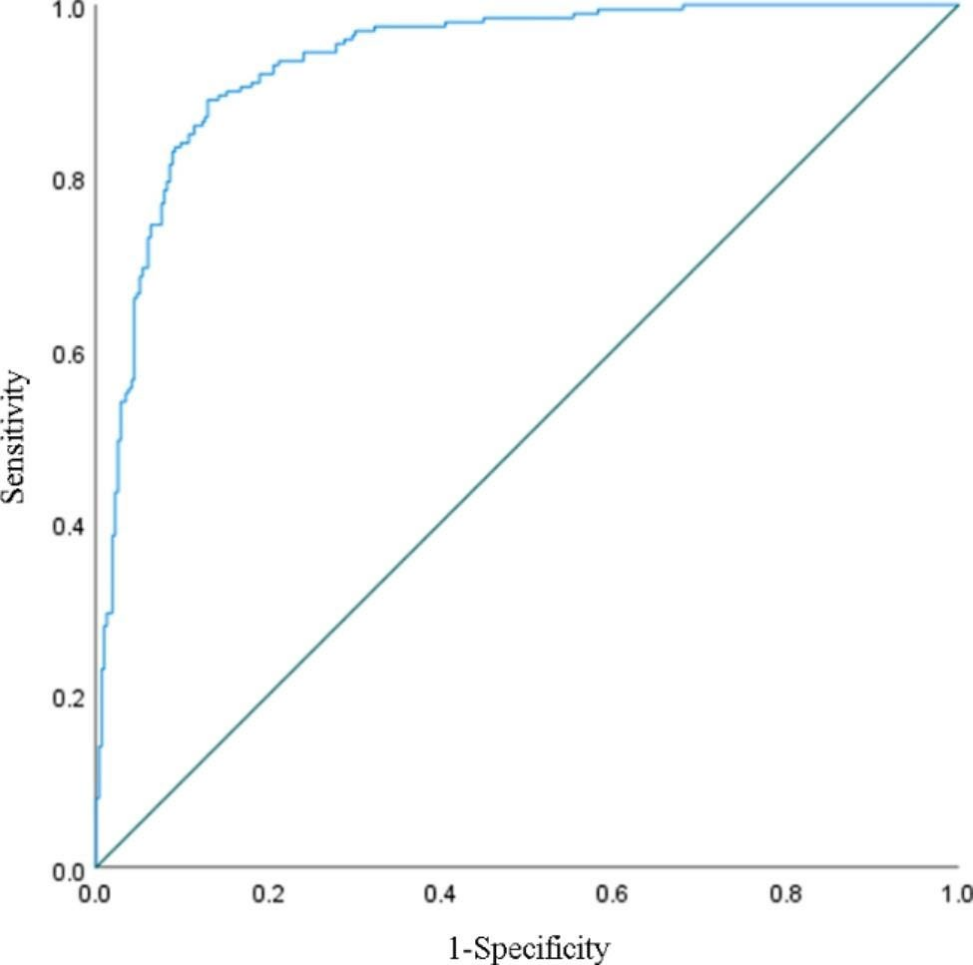
ROC curve when three tumor markers in combination with MPV/PC for the diagnosis of CRC.

Next, we assessed the efficacy of the combination of MPV/PC with tumor markers in the diagnosis of CRC. Through the ROC curve, we found that the AUC, sensitivity, specificity and Youden index of MPV/PC in combination with the three markers was respectively 0.936, 0.890, 0.870 and 0.760, all higher than that of the four indexes used alone for diagnosing CRC. In addition, we also found that the PPV and NPV of the combination of the three tumor markers with MPV/PC were higher than the tumor markers or MPV/PC used alone in the diagnosis of CRC (Table 4; Fig. 3). Therefore, we concluded that the combination of MPV/PC with the three tumor markers has higher efficacy in the diagnosis of CRC.

**Table 4:** Diagnostic performances of the three tumor markers in combination with MPV/PC for distinguishing colorectal cancer from benign colorectal polyps

Items	AUC(95%CI)	sensitivity	specificity	Youden index	PPV	NPV
CA72-4 + CA125 + CA199 + MPV/PC	0.936(0.916–0.957)	0.890	0.870	0.760	0.812	0.926

## Discussion

In addition to reflecting the platelet size and activity, MPV is also an inflammatory marker in cardiovascular and rheumatologic diseases [19]. Although MPV has been identified as an early diagnostic marker in the detection of multiple cancers, the effect of MPV on the progression of tumors remains ambiguous. Elevated MPV is associated with a better outcome in patients with breast cancer [20] and worse overall survival in patients with CRC [21]. However, a prospective clinical study exploring the correlation between MPV and prognosis of patients treated with chemotherapy regimens such as FOLFOX/XELOX/FOLFIRI demonstrated that decreased MPV level in patients receiving chemotherapy was associated with worse overall survival compared with the elevated MPV level group [22]. Our study showed that the MPV level was lower in the CRC group than in the adenomatous polyp group, corresponding to the results of Lalosevic et al. [11].

Platelets are non-nucleated cells associated with inflammation and thrombosis, and cytokines such as platelet-derived growth factor and vascular endothelial growth factor produced by tumor cells can promote the activity and production of platelet [13]. Due to the high response to inflammation, a significant proportion of larger platelets are degraded under inflammatory conditions and ultimately lead to a decrease in MPV [23]. Previous studies indicated that the ratio of MPV to PC should be interpreted as a ratio rather than being used alone [16]. Recently, numerous studies have focused on the MPV/PC ratio in the diagnosis and prognosis of malignancies. Cho et al. reported for the first time a higher sensitivity and specificity of MPV/PC in the diagnosis of hepatocellular carcinoma than MPV alone [24]. A low MPV/PC ratio is more valuable than MPV or PC alone in predicting the prognosis of patients with non-small cell lung cancer [25]. MPV/PC was significantly lower in patients with esophageal squamous cell carcinoma than in the control group, and a lower MPV/PC was associated with local progression and a worse prognosis [23]. Similarly, a lower MPV/PC was associated with poor prognosis in patients with gastric cancer, and MPV/PC has a higher accuracy than NLR or PLR in predicting the prognosis of gastric cancer [26].

Consistent with the previous studies, we found that MPV/PC was significantly lower in CRC patients than in the control group. MPV/PC showed no profound difference in the distant metastasis subgroup. However, preoperative MPV/PC was significantly lower in patients with CRC of T3-4 stage, lymph node metastasis, poorly differentiated or nerve invasion. When CA72-4, CA125, CA199 and MPV/PC were used alone to diagnose CRC, the AUC, sensitivity, specificity and Youden index of CA72-4 was the highest. However, when MPV/PC was combined with the three tumor markers, the AUC, sensitivity, specificity and Youden index of the combination were higher than when the three tumor markers or MPV/PC was used alone, indicating that a combination of MPV/PC with the three tumor markers had a better efficacy for the diagnosis of CRC.

Mounting evidence suggests that systemic inflammation participates in various stages of tumor development through multiple mechanisms [27, 28]. Chronic inflammatory bowel disease increased the risk of CRC without the classical adenoma-carcinoma transformation process by acting as the trigger of choric inflammation [29]. Furthermore, in support of that assumption, another study indicated that non-steroidal anti-inflammatory drug application reduced the systemic inflammation and risk of CRC [30], suggesting that systemic inflammation is extremely significant in CRC development. Most CRCs develop through the adenoma-carcinoma sequence, and the progression is relatively slow (10 to 15 years) [31], presenting opportunities to prevent cancer by removing its precursor lesions, in addition to identifying CRC in its earliest, curable stages. Relevant studies suggest that the earlier CRC or precancerous lesions are detected, the higher the survival rate of patients [32]. Yet, the majority of CRC is sporadic and largely attributable to the constellation of modifiable environmental risk factors characterizing westernization (for example, obesity, physical inactivity, poor diets, et al.). The risk factors for CRC persist, but effective ways to reduce risk factors and systematic interventions are absent. On the other hand, the incidence of CRC in patients aged 50 years is increasing. The American Cancer Society estimated that 11.0% of colon cancers and 14.7% of rectal cancers in 2020 were diagnosed in people aged 50 years [33]. Therefore, the early screening of CRC is of great significance. For the elderly population or the lower income groups or countries, the standard screening stool colonoscopy may do more harm than good. Therefore, the combination of MPV/PC and tumor markers provides a novel option for the early screening of CRC.

In our study, we found that MPV/PC was associated with indicators associated with malignant behavior (large tumor size, lymph node metastasis, poorly differentiated or nerve invasion, etc.). Therefore, we guessed that the combination of MPV/PC with tumor markers may act as a prognosis prediction index. In advanced non-small-cell lung cancer, a low MPV/PC ratio may be correlated with increased monocytic myeloid-derived suppressor cells which represent one of the key mechanisms in immunosuppressive tumor microenvironments to play major roles not only in the carcinogenesis of lung cancer but also in disease progression and prognosis and, in addition, predict the efficacy of immune checkpoint inhibitors [34]. Tumor mismatch repair defect in CRC may lead to high tumor mutation burden and more tumor neoantigens are produced, thus influencing the tumor microenvironment to infiltrate more T cells required for antitumor immune activation, and therefore, potentially achieving better immunotherapeutic efficacy [35]. Thus, we speculated that the value of MPV/PC also may indicate certain connection between MPV/PC and immunotherapy in CRC. Of course, the progress prediction value, the association between MPV/PC and immunotherapy and the underline mechanism need to be further confirmed by future prospective studies and basic researches. Colon and rectal cancer differ in epidemiology, genetics, molecular, morbidity and prognosis. Both left-sided and right-sided CRC patients with low MPV levels tended to have shorter DFS [18]. We hence guessed that MPV/PC may also play a role in the progress prediction of colon and rectal cancer, and the combination of MPV/PC and tumor markers may show different diagnostic efficacy between colon and rectal cancer. Of course, the above hypotheses all need to be further investigated.

Several studies have investigated the value of MPV/PC in the diagnosis and progression prediction of malignancies, and tumor markers such as CA72-4, CA125 and CA199 are commonly used in diagnosis, treatment efficacy evaluation and recurrence monitoring. However,only a few studies have investigated the value of the combination of MPV/PC with tumor markers in CRC. To the best of our knowledge, this is the first study to investigate the clinical value of the combination of MPV/PC with tumor markers in CRC. The high sensitivity and specificity of the combination suggest that MPV/PC may be a promising novel marker for early diagnosis of CRC.

Nonetheless, this study has some limitations. First, relatively few cases were enrolled, and therefore our conclusions should be further validated using large-scale multicenter clinical studies. Second, this is a retrospective study lacking longitudinal observational data, and hence it cannot completely resolve some confounding factors and may produce a certain degree of deviation. Finally, due to limited data, the study failed to investigate the influence of treatment on MPV/PC change and the correlation between CRC progression and MPV/PC, therefore, further prospective studies is expected to be carried out in our following study to explore the prognostic value of MPV/PC in patients undergoing primary surgery by following for recurrence, metastasis and survival. Overall, limitations notwithstanding, the present study has highlighted the significance of MPV/PC on CRC and provided a reference value for the early diagnosis and prognosis of CRC to some extent.

## Data Availability

The datasets used and/or analyzed during the current study are available from the corresponding author on reasonable request. The data presented in this study are available on request from the corresponding author. The data are not publicly available.

## Abbreviations

CRC: Colorectal cancer
AUC (95% CI): Area under the receiver operating characteristic curve (95% confidence interval)
MPV: Mean platelet volume
MPV/PC: Ratio between MPV and platelet count
NLR: Neutrophil to lymphocyte ratio
PC: Platelet count
PLR: Platelet to lymphocyte ratio
ROC: Receiver operating characteristic.
